# Global, regional and national burden of deciduous dental caries from 1990 to 2021: analysis of risk factors and prediction of trends in 2035

**DOI:** 10.3389/fdmed.2025.1624571

**Published:** 2025-10-07

**Authors:** Hong Yang, Yong Feng, Li-Yuan Xiao, Kai-Mei Wang, Hong-Chao Feng

**Affiliations:** ^1^ Department of Prosthodontics, Guiyang Hospital of Stomatology, Guiyang, China; ^2^ ZBH-Center for Bioinformatics, Universität Hamburg, Hamburg, Germany; ^3^ Department of Oral and Maxillofacial Surgery, Division of Regenerative Orofacial Medicine, University Medical Center Hamburg-Eppendorf, Hamburg, Germany

**Keywords:** deciduous dental caries, global burden of disease, socio-demographic index, ARIMA model, public health

## Abstract

**Background/aim:**

Early childhood caries (ECC) is one of the most prevalent global oral health issues in children under five years old, significantly impacting their overall health and quality of life. This study aimed to analyze the global, regional, and national burden of ECC from 1990 to 2021, identify associated risk factors, and predict trends through 2040. It further examined the influence of socio-demographic factors, sex, and age on ECC incidence and prevalence.

**Patients and methods:**

Data from the Global Burden of Disease (GBD) 2021 database were analyzed using Bayesian Meta-Regression models to estimate ECC incidence, prevalence, and years lived with disability (YLDs) across 204 countries and regions. Socio-Demographic Index (SDI) levels, sex, and age-specific trends were assessed. Age-standardized rates and estimated annual percentage changes (EAPCs) were calculated. Predictive models, including ARIMA, were developed to forecast future trends.

**Results:**

Between 1990 and 2021, ECC incidence and prevalence showed modest declines globally, with significant variations across SDI regions. High SDI regions exhibited the lowest burden due to effective public health measures, while medium and low SDI regions faced persistent challenges. Sex disparities were observed, with males showing slightly higher rates than females. Children aged 5–9 years bore the highest burden of ECC. Predictions for 2040 indicate a potential rebound in ECC incidence in low and medium SDI regions without targeted interventions.

**Conclusion:**

This study highlights the substantial burden of ECC, particularly in under-resourced regions, and underscores the need for tailored public health strategies. Effective prevention, improved healthcare access, and education are critical to reducing ECC burden globally, ensuring better oral health outcomes for children, and addressing disparities across socio-economic, sex, and age groups.

## Introduction

Early childhood caries (ECC), or early childhood tooth decay, is one of the most prevalent oral diseases in children under the age of five worldwide. ECC has complex etiologies linked to multiple factors, including oral hygiene practices, dietary habits, socioeconomic status, and parental health awareness (1). Typical characteristics of ECC include enamel and dentin demineralization and softening, which can lead to tooth breakage and even loss, impacting chewing function, language development, nutrient absorption, and children's quality of life (2). If left untreated, ECC can progress to severe pulp infections and periradicular lesions, resulting in pain and potentially leading to systemic illnesses (3). Thus, ECC is not only an oral health issue but also a significant public health concern affecting children's overall health.

Data from the 2019 and 2021 Global Burden of Disease (GBD) studies indicate a continued increase in the global burden of ECC, especially in low- and middle-income countries (LMICs) (4). More than 500 million children are estimated to be affected by ECC worldwide, with prevalence significantly higher in developing countries and impoverished regions than in high-income nations (5). Research also suggests that childhood oral health is closely related to overall health status in adulthood (6). Therefore, ECC prevention and treatment are not only vital for childhood health but may also have long-term implications for adult health. Despite this, awareness and prevention of ECC remain insufficient globally, particularly in low- and lower-middle-income countries, where children's oral health is often overlooked, and resources for health services and education are severely lacking (7).

The pathogenesis of ECC involves multiple factors, including bacteria (such as Streptococcus mutans), dietary influences (such as frequent intake of sugary foods and drinks) (8), poor oral hygiene (such as irregular tooth brushing), socioeconomic factors (such as household income and parental education), and cultural practices (9–11). Additionally, studies have demonstrated correlations between ECC and children's nutritional status, body mass index (BMI), and cognitive development (12). The severity of ECC can negatively impact children's physical and mental development, potentially leading to malnutrition, stunted growth, and attention deficits (13). Therefore, developing effective public health policies, raising health awareness among parents and children, and improving access to oral health services—especially in LMICs—are crucial strategies for controlling ECC burden (14, 15).

Although many studies have explored the causes and control strategies of ECC, there remains a lack of systematic research on the epidemiological characteristics and burden trends of ECC globally (16). Studies based on the GBD database provide crucial data for analyzing ECC burden at global and regional levels; however, data on ECC burden distribution and trend changes across different Socio-Demographic Index (SDI) regions, sexes, and age groups remain unclear. Thus, a comprehensive global study is urgently needed to address this gap (17).

This study, based on the GBD 2021 database, aimed to systematically assess ECC burden worldwide and across regions with varying SDI levels from 1990 to 2021, examine temporal trends, and analyze the relationship between ECC burden, SDI, sex, and age distribution. By analyzing long-term dynamic changes in the global ECC burden, this study seeks to provide scientific evidence for formulating targeted public health policies and oral health interventions across regions. Additionally, the study uses predictive models to project ECC burden trends for the coming decades, offering guidance for public health policymakers to effectively control ECC burden, improve global children's oral health, and enhance overall quality of life.

## Materials and methods

This study assessed the burden of early childhood caries (ECC) globally and across regions with different SDI levels from 1990 to 2021 using data from the GBD database. A systematic approach was applied to data collection, model estimation, and statistical analysis, as follows:

### Data source

Data were sourced from the GBD 2021 database on the Global Health Data Exchange platform (GHDx), covering ECC epidemiological data from 1990 to 2021 for 204 countries and territories (18). ECC burden was evaluated using metrics such as prevalence, incidence, and years lived with disability (YLDs) across global and SDI regions. Data were stratified by sex (male and female) and age groups (<1 year, 2–4 years, 5–9 years, 10–14 years) and categorized according to SDI levels of different regions and countries.

### Case definition and disease model

The case definition for ECC was based on the International Classification of Diseases, 10th Revision (ICD−10) standard, using code K02.3. The study population included children under five years of age (including those in the 2–4 and 5–9 age groups) with ECC from 1990 to 2021. All disease modeling was conducted using the Bayesian Meta-Regression tool, DisMod-MR, developed under the GBD framework. DisMod-MR is a Bayesian stratified model that synthesizes and estimates data across studies in the absence of direct observational data. This model integrates prevalence, incidence, and burden data from multiple sources, using Monte Carlo simulations to estimate global and regional disease burden, providing 95% uncertainty intervals (UI).

### Data analysis and processing

#### Calculation of epidemiological metrics

The age-standardized prevalence rate, incidence rate, and disability-adjusted life years (DALYs) for ECC were calculated following GBD standardized methods. Descriptive and trend analysis assessed long-term changes in ECC burden at global and regional levels. All analyses used the age-standardized rate (ASR) and EAPC to represent the yearly trends of epidemiological metrics. Countries and regions were divided into five categories by SDI level: low SDI (<0.46), low-middle SDI (0.46–0.60), middle SDI (0.61–0.69), high-middle SDI (0.70–0.81), and high SDI (>0.81), allowing comparisons of ECC burden across development levels.

### Analysis of regional disparities and SDI correlation

Regression models were used to examine the relationship between SDI and ECC burden. By calculating the EAPC for each country and region, trends in ECC burden across various SDI levels were analyzed. To further explore the SDI-ECC burden relationship, a generalized additive model (GAM) was used to evaluate ECC burden relative to SDI, with a scatter plot generated for EAPC and SDI.

### Prediction model development

An Autoregressive Integrated Moving Average (ARIMA) model was used to estimate ECC burden trends through 2040 at global and SDI-specific levels. Age-standardized prevalence, incidence rates, and YLDs from 1990 to 2021 were used as independent variables, with SDI level as a key covariate. Model estimates were based on 1,000 Monte Carlo simulations, with the 95%UI defined between the 25th and 975th simulations.

### Statistical methods

All data analyses were conducted using R software (v4.3.3) following the Guidelines for Accurate and Transparent Health Estimates Reporting (GATHER). Descriptive statistics were used to assess sample characteristics, and EAPC for all epidemiological indicators was calculated using a linear regression model, with statistical significance set at *p* < 0.05. Sensitivity analyses ensured the robustness and reliability of estimates, and ECC burden trends were visualized with line graphs, heatmaps, and scatter plots for global, regional, and country-specific data.

### Ethical considerations

The data used in this study were obtained from the publicly available GBD database, with all data anonymized, thus exempting this study from ethical approval. Additionally, the study adhered to the GBD Study Framework and GATHER guidelines to ensure transparency in data usage, accuracy of results, and reproducibility of methods.

## Results

### Global incidence and prevalence of dental caries in primary teeth

In 2021, the global incidence and prevalence of dental caries in primary teeth showed slight differences between males and females, with males having a slightly higher incidence rate. Specifically, the age-standardized incidence rate (ASR) for males was 17,921.72 per 100,000 population (95% uncertainty interval: 14,075.56–23,235.69), compared to 17,631.42 per 100,000 for females (95% uncertainty interval=13,833.09–22,759.74). The age-standardized years lived with disability rate (ASYR) was similar between males and females, at 2.90 and 2.87, respectively (Table 1).

**Table 1:** Overview of the incidence, prevalence, and years lived with disability (YLD) rates for deciduous caries by sex globally and in SDI regions in 2021, along with the corresponding age-standardized rates and estimated annual percentage change (EAPC) values.

SDI level	2021	ASR (per 100,000)	2021	ASR (per 100,000)	2021	ASR (per 100,000)	EAPC 1990–2021		
	Incidence		Prevalence		YLDs		ASIR	ASPR	ASYR
Global									
Both	1253255083.96 (978655131.44 to 1637292359.08)	17781.15 (13952.4 to 23035.44)	524630097.18 (437684569.6 to 611230119.17)	7548.04 (6290.61 to 8775.83)	200728.18 (88103.16 to 385501.9)	2.89 (1.27 to 5.54)	−0.27% (−0.30% to −0.24%)	−0.25% (−0.27% to −0.22%)	−0.24% (−0.26% to −0.22%)
Female	601336674.15 (468390011.9 to 783417863.43)	17631.42 (13833.09 to 22759.74)	252405818.59 (210638983.01 to 294133095.52)	7504 (6256.33 to 8722.96)	96567.52 (42393.37 to 185626.84)	2.87 (1.26 to 5.51)	−0.27% (−0.31% to −0.23%)	−0.24% (−0.27% to −0.22%)	−0.24% (−0.26% to −0.21%)
Male	651918409.81 (509508199.5 to 852356427.2)	17921.72 (14075.56 to 23235.69)	272224278.59 (226729241.74 to 316820288.48)	7589.28 (6313.3 to 8811.21)	104160.66 (45647.88 to 199875.07)	2.9 (1.27 to 5.56)	−0.26% (−0.29% to −0.23%)	−0.25% (−0.28% to −0.23%)	−0.24% (−0.27% to −0.21%)
Low SDI									
Both	268462866.4 (211181817.01 to 337270894.77)	16807.83 (13200.69 to 21158.83)	114201376.33 (94671075.08 to 132399780.9)	7116.18 (5899.87 to 8255.52)	43561.7 (19564.76 to 83140.18)	2.71 (1.22 to 5.19)	−0.58% (−0.66% to −0.51%)	−0.41% (−0.45% to −0.38%)	−0.40% (−0.44% to −0.36%)
Female	130259142.45 (102153928.48 to 162483293.94)	16618 (13016.3 to 20759.64)	55654682 (46186150.23 to 64508592.61)	7072.57 (5871.01 to 8195.62)	21231.87 (9662.7 to 40442.02)	2.7 (1.23 to 5.14)	−0.57% (−0.66% to −0.49%)	−0.41% (−0.44% to −0.37%)	−0.39% (−0.43% to −0.36%)
Male	138203723.95 (108487036.33 to 175231238.3)	16991.4 (13321.36 to 21615.57)	58546694.34 (48317481.36 to 68040748.92)	7158.03 (5903.72 to 8319.98)	22329.82 (9895.33 to 42769.77)	2.73 (1.21 to 5.24)	−0.59% (−0.68% to −0.51%)	−0.42% (−0.47% to −0.37%)	−0.40% (−0.46% to −0.35%)
Low-middle SDI									
Both	347437185.3 (270686475.18 to 441126496.47)	17293.08 (13537.65 to 21823.29)	145612625.77 (120572398.79 to 169096002.68)	7340.64 (6072.71 to 8511.13)	55673.35 (25189.55 to 106976.17)	2.81 (1.27 to 5.39)	−0.38% (−0.42% to −0.33%)	−0.31% (−0.34% to −0.27%)	−0.29% (−0.32% to −0.26%)
Female	167178499.9 (129530769.47 to 212685730.66)	17153.23 (13358.41 to 21689.37)	69998227 (57991444.88 to 81236625.19)	7272.36 (6022.29 to 8442.64)	26773.41 (12098.5 to 51435.02)	2.78 (1.26 to 5.34)	−0.39% (−0.45% to −0.34%)	−0.31% (−0.34% to −0.28%)	−0.29% (−0.32% to −0.26%)
Male	180258685.39 (140946492.78 to 229885415.12)	17424.95 (13677.48 to 21963.79)	75614398.78 (62530786.42 to 87951029.98)	7404.87 (6117.39 to 8610.66)	28899.94 (13059 to 55572.72)	2.83 (1.28 to 5.44)	−0.36% (−0.42% to −0.31%)	−0.31% (−0.35% to −0.27%)	−0.29% (−0.33% to −0.25%)
Middle SDI									
Both	375160882.52 (287550074.2 to 507765420.45)	18716.08 (14560.23 to 24984.64)	161792754.78 (136502510.34 to 188810906.08)	8267.38 (6978.71 to 9613.96)	61977.9 (26913.53 to 118731.57)	3.17 (1.38 to 6.05)	−0.09% (−0.13% to −0.06%)	−0.06% (−0.07% to −0.05%)	−0.05% (−0.06% to −0.04%)
Female	178587274.36 (137488923.15 to 240098712.61)	18579.89 (14463.94 to 24655.29)	77487547.22 (65316884.7 to 90233820)	8256.83 (6968.1 to 9579.56)	29668.35 (12952.74 to 56636.13)	3.16 (1.38 to 6.02)	−0.09% (−0.12% to −0.05%)	−0.05% (−0.06% to −0.04%)	−0.04% (−0.05% to −0.03%)
Male	196573608.17 (149710867.03 to 267585110.59)	18841.74 (14520.61 to 25288.54)	84305207.56 (71034838.36 to 98844597.91)	8277.05 (6981.71 to 9665.41)	32309.55 (14018.06 to 62130.07)	3.17 (1.38 to 6.08)	−0.10% (−0.14% to −0.07%)	−0.06% (−0.08% to −0.05%)	−0.05% (−0.07% to −0.04%)
High-middle SDI									
Both	160772187.35 (119783532.22 to 223414084.31)	19383.69 (14609.16 to 26602.53)	65591744.9 (54050719.15 to 78721334.94)	8132.91 (6697.29 to 9703.56)	25156.56 (10771.32 to 49712.73)	3.12 (1.34 to 6.15)	−0.07% (−0.08% to −0.06%)	−0.12% (−0.14% to −0.10%)	−0.11% (−0.13% to −0.09%)
Female	76386674.89 (56603603.49 to 105947306.32)	19339.12 (14513.54 to 26489.26)	31258551.96 (25785413.59 to 37472408.75)	8140.45 (6719.41 to 9686.98)	11989.45 (5125.43 to 23759.94)	3.12 (1.35 to 6.17)	−0.07% (−0.08% to −0.05%)	−0.11% (−0.13% to −0.09%)	−0.11% (−0.13% to −0.09%)
Male	84385512.46 (63057674.91 to 117466778)	19424.39 (14587.72 to 26705.9)	34333192.94 (28264908.32 to 41111099.6)	8126.06 (6689.93 to 9714.65)	13167.11 (5648.06 to 25952.8)	3.12 (1.34 to 6.13)	−0.08% (−0.09% to −0.06%)	−0.13% (−0.15% to −0.11%)	−0.12% (−0.14% to −0.10%)
High SDI									
Both	100458883.66 (76316892.82 to 131720471.66)	16609.59 (12657.62 to 21501.69)	37012428.38 (30295749.05 to 44709743.75)	6257.88 (5147.34 to 7530.88)	14198.15 (5972 to 27949.32)	2.4 (1.01 to 4.7)	−0.10% (−0.17% to −0.02%)	−0.44% (−0.63% to −0.26%)	−0.44% (−0.63% to −0.25%)
Female	48459002.74 (36834819.92 to 63494324.88)	16457.5 (12606.51 to 21360.53)	17803286.35 (14626320.7 to 21616168.73)	6179.6 (5097.34 to 7471.4)	6826.53 (2882.06 to 13566.03)	2.37 (1 to 4.69)	−0.09% (−0.16% to −0.02%)	−0.40% (−0.59% to −0.21%)	−0.40% (−0.59% to −0.21%)
Male	51999880.92 (39302293.13 to 67947710.99)	16753.86 (12728.42 to 21665.82)	19209142.03 (15678403.4 to 23131015.99)	6332.19 (5202.79 to 7606.65)	7371.63 (3089.94 to 14383.3)	2.43 (1.02 to 4.72)	−0.11% (−0.19% to −0.03%)	−0.48% (−0.66% to −0.30%)	−0.48% (−0.66% to −0.29%)

Values in parentheses represent 95% uncertainty intervals.

ASIR, age-standardized incidence rate; ASPR, age-standardized prevalence rate; ASYR, age-standardized YLD rate; ASR, age-standardized rate (per 100,000 population); SDI, socio-demographic index.

EAPC data indicate a declining trend in the incidence, prevalence, and ASYR of dental caries in primary teeth from 1990 to 2021. The EAPC for incidence was −0.27% (males −0.26%, females −0.27%), for prevalence it was −0.25% (males −0.25%, females −0.24%), and for ASYR it was −0.24% for both males and females. These data suggest that, although the global burden of dental caries in primary teeth has decreased, the reduction has been modest.

### Differences in dental caries in primary teeth across SDI regions

Significant differences were observed in the epidemiology of dental caries in primary teeth across different SDI regions:

#### Low SDI region

The incidence rate and ASYR were relatively low in low SDI regions, but the EAPC showed a significant decline, particularly in males, whose incidence rate declined by −0.59%. This suggests that, despite limited resources, some effective interventions were implemented to control dental caries in primary teeth in these regions.

#### Medium SDI region

Medium SDI regions (including low-middle and medium SDI) showed higher incidence and prevalence rates, with relatively stable EAPC changes. In the low-middle SDI region, the ASR for males was 17,424.95 per 100,000, compared to 17,153.23 per 100,000 for females. This indicates a continued challenge in managing dental caries effectively in medium SDI regions.

#### High SDI region

High SDI regions had the lowest burden of dental caries in primary teeth, with both incidence and prevalence rates significantly lower than those in other regions. The EAPC for ASYR was −0.44%, indicating significant success in controlling dental caries in these regions through effective public health interventions.

### Sex differences

The incidence and prevalence rates of dental caries in primary teeth were generally higher in males compared to females, although the ASYR showed no significant sex difference. This disparity may be attributed to differences in caries pathogenesis, dietary habits, and oral hygiene practices between males and females, which require further investigation to elucidate potential mechanisms.

### Age and sex characteristics of dental caries in primary teeth

#### Incidence

**Figure 1:**
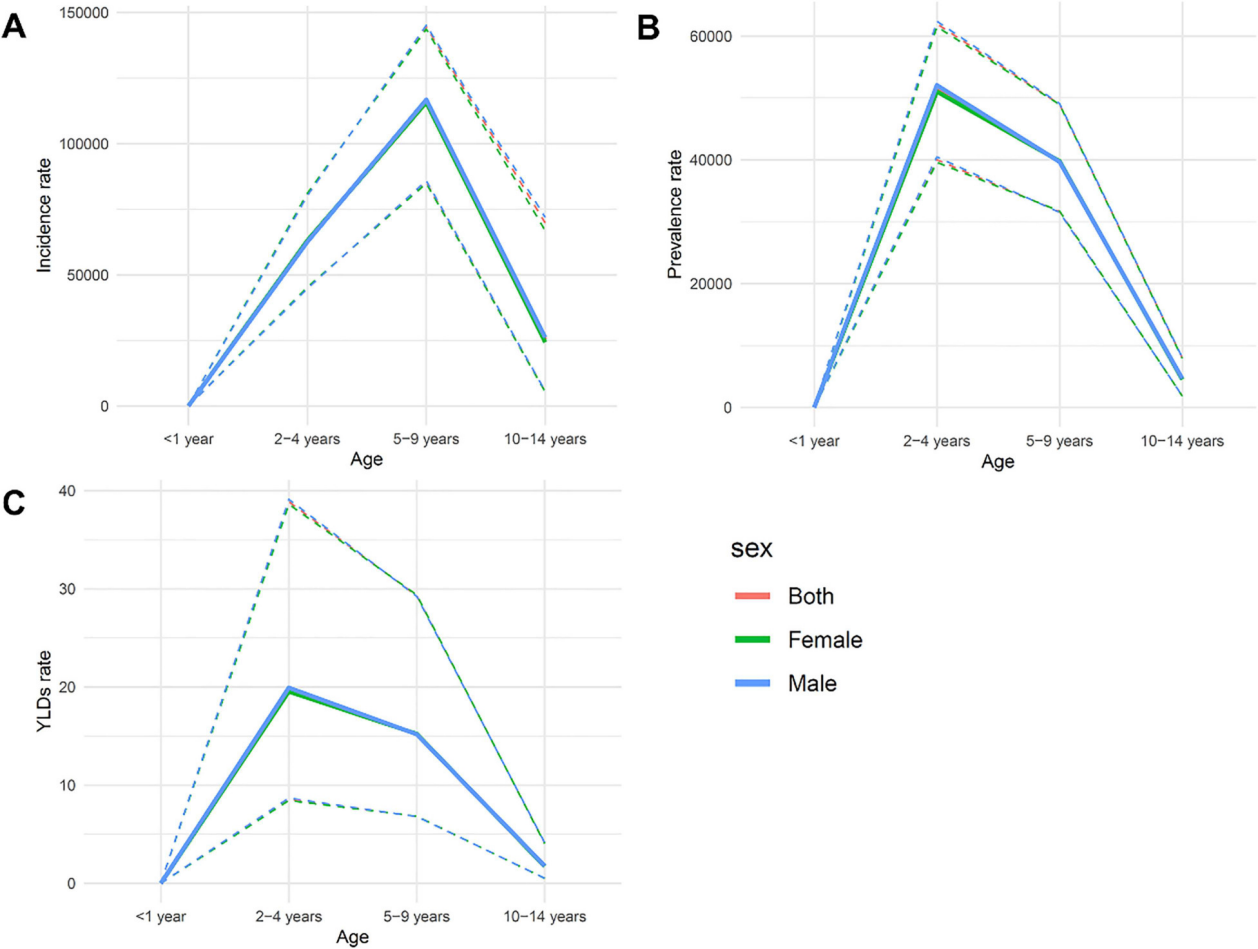
The incidence **(A)**, prevalence **(B)**, and ASYR **(C)** of dental caries in primary teeth according to age and sex in 2021.

The incidence of dental caries in primary teeth increased markedly among children aged 2–9 years, peaking at ages 5–9. This peak may be associated with the complete eruption of primary teeth and challenges in maintaining oral hygiene during this period. Data showed that the incidence rate was slightly higher in males than in females, suggesting subtle sex-related differences in oral health behaviors or exposure to risk factors (Figure 1A).

#### Prevalence

The prevalence of dental caries followed a similar pattern to incidence, increasing significantly between ages 2 and 9 and peaking at ages 5–9, then declining in the 10–14 age group. This indicates that the peak burden of dental caries occurs during the preschool and early school years, likely influenced by changing dietary habits and lower compliance with oral hygiene practices. Additionally, the prevalence was consistently higher in males, reinforcing the trend observed in incidence (Figure 1B).

#### ASYR

The ASYR peaked among children aged 2–4 years and gradually declined between ages 5 and 14. Notably, ASYR was higher in males across all age groups, with the greatest difference seen in the 5–9 age group. This difference may be attributed to male behavioral factors, such as less frequent oral hygiene practices or poorer dietary choices (Figure 1C).

The incidence, prevalence, and ASYR of dental caries in primary teeth showed significant differences across age groups and sex. Ages 2–9 were the most burdened, particularly the 5–9 age group, which should be a focal point for public health interventions aimed at reducing the burden of dental caries in this population. Sex differences should also be taken into consideration when formulating policies, including health education and preventive measures specifically targeting boys to improve oral health behaviors.

### Trends in dental caries in primary teeth across SDI regions

#### Age-standardized incidence rate

**Figure 2:**
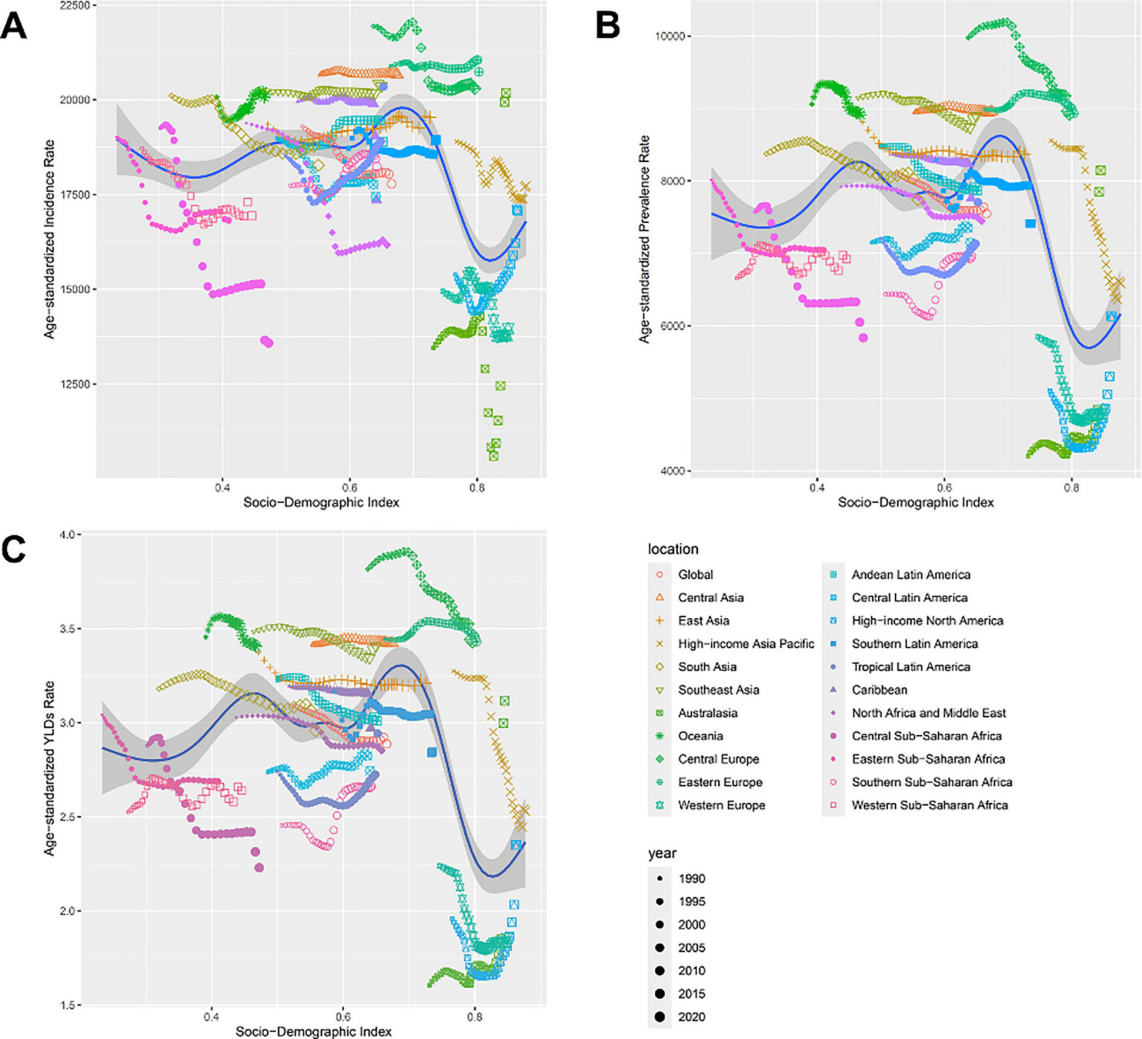
Illustrates that high-income Asia-Pacific and Western Europe generally have lower rates, whereas central and east Asia display higher rates. **(A)** Age-standardized incidence, **(B)** prevalence, and **(C)** YLD rates of oral disorders across the socio-demographic index (SDI).

The incidence rate showed significant variation across SDI levels, with an initial increase followed by a decrease. As the SDI increased, the incidence rate peaked in medium SDI regions and then slightly declined, reflecting greater susceptibility to dental caries in medium SDI regions due to socio-economic conditions. High SDI regions benefited from improved oral health measures and awareness, resulting in lower incidence rates (Figure 2A).

#### Age-standardized prevalence rate

The prevalence rate followed a similar trend to the incidence rate, peaking in medium SDI regions before declining. Medium SDI regions still face challenges in caries prevention, highlighting gaps in public health infrastructure (Figure 2B).

#### Age-standardized YLD rate

The ASYR was higher in low and medium SDI regions and gradually decreased in high SDI regions, indicating that socio-economic development helps reduce the long-term disease burden of dental caries (Figure 2C).

### Epidemiological differences across regions and sex

#### Central Asia and East Asia

Both regions had relatively high incidence and prevalence rates. The ASR for Central Asia was 20,660.8 per 100,000, while in East Asia it was 19,535.58 per 100,000. However, East Asia showed a slightly lower ASYR, indicating some progress in disease management. Notably, East Asia exhibited a slight increase in incidence (EAPC of 0.09%) but a decline in prevalence (EAPC of −0.09%), reflecting mixed progress (Table 2).

**Table 2:** An overview of the incidence, prevalence, and years lived with disability (YLD) rates for deciduous caries by sex in regions in 2021, along with the corresponding age-standardized rates and estimated annual percentage change (EAPC) values.

Area	2021	ASR (per 100,000)	2021	ASR (per 100,000)	2021	ASR (per 100,000)	EAPC 1990–2021		
	Incidence		Prevalence		YLDs		ASIR	ASPR	ASYR
Central Asia									
Both	20073775.69 (14853259.47 to 28006955.22)	20660.8 (15171.16 to 29103.16)	8783221.52 (7330690.11 to 10474578.2)	8950.71 (7456.7 to 10685.24)	3358.41 (1454.07 to 6601.42)	3.42 (1.48 to 6.73)	−0.01% (−0.01% to 0.00%)	0.00% (−0.01% to 0.01%)	0.01% (0.00% to 0.02%)
Female	9650084.88 (7099737.33 to 13457837.98)	20603.69 (15075.43 to 29002.5)	4254646.37 (3539008.24 to 5095929.99)	8996.69 (7467.26 to 10798.11)	1625.21 (703.08 to 3201.35)	3.44 (1.49 to 6.77)	−0.01% (−0.02% to 0.00%)	0.00% (−0.01% to 0.01%)	0.01% (0.00% to 0.02%)
Male	10423690.8 (7706131.94 to 14549117.24)	20713.93 (15210.33 to 29196.81)	4528575.15 (3778688.11 to 5396324.54)	8907.92 (7427.39 to 10639.18)	1733.2 (745.16 to 3431.86)	3.41 (1.47 to 6.76)	0.00% (−0.01% to 0.01%)	−0.01% (−0.02% to 0.01%)	0.01% (−0.01% to 0.02%)
East Asia									
Both	191229653.59 (143726443.6 to 259783009.99)	19535.58 (14782.72 to 26418.79)	80092912.56 (66473914.81 to 94914838.16)	8371.49 (6968.42 to 9868.18)	30730.82 (13288.75 to 61033.93)	3.21 (1.39 to 6.37)	0.09% (0.06% to 0.11%)	−0.09% (−0.13% to −0.05%)	−0.08% (−0.12% to −0.05%)
Female	89465219.72 (67614135.38 to 121706452.53)	19548.95 (14872.12 to 26472.59)	37645677.97 (31293821.88 to 44334285.82)	8426.12 (7019.76 to 9897.1)	14442.97 (6272.79 to 28580.05)	3.23 (1.41 to 6.39)	0.11% (0.09% to 0.13%)	−0.07% (−0.11% to −0.03%)	−0.07% (−0.11% to −0.03%)
Male	101764433.87 (76258719.4 to 138228080.67)	19524.28 (14721.43 to 26371.72)	42447234.59 (35202287.2 to 50641236.17)	8323.8 (6907.91 to 9871.25)	16287.86 (7031.26 to 32461.65)	3.19 (1.38 to 6.36)	0.06% (0.03% to 0.10%)	−0.10% (−0.14% to −0.06%)	−0.09% (−0.13% to −0.06%)
High-income Asia Pacific									
Both	13991082.23 (10968936.92 to 18085864.12)	17734.54 (14055.47 to 22399.67)	4987696.85 (4181536.37 to 5768841.47)	6583.77 (5538.7 to 7585.11)	1916.13 (854.66 to 3598.92)	2.53 (1.13 to 4.71)	−0.24% (−0.29% to −0.19%)	−1.10% (−1.20% to −0.99%)	−1.09% (−1.19% to −0.98%)
Female	6835398.6 (5356326.99 to 8783438.47)	17767.39 (14113.17 to 22369.14)	2413189.22 (2030314.55 to 2799655.92)	6530.76 (5482.19 to 7555.85)	927.14 (413.36 to 1745.58)	2.51 (1.13 to 4.68)	−0.22% (−0.27% to −0.17%)	−1.11% (−1.20% to −1.02%)	−1.10% (−1.19% to −1.01%)
Male	7155683.64 (5573018.48 to 9279840.99)	17703.32 (13982.67 to 22467.19)	2574507.63 (2162064.23 to 2970103.37)	6634.19 (5587.11 to 7624.18)	989 (442.26 to 1853.34)	2.55 (1.14 to 4.76)	−0.26% (−0.32% to −0.20%)	−1.09% (−1.21% to −0.97%)	−1.07% (−1.19% to −0.96%)
South Asia									
Both	306228969.49 (237842531.77 to 400194289.08)	17488.24 (13708.08 to 22367.38)	131969022.17 (109996813.71 to 152944235.86)	7743.24 (6450.26 to 8999.56)	50409.43 (22618.99 to 96805.74)	2.96 (1.33 to 5.65)	−0.36% (−0.41% to −0.31%)	−0.23% (−0.28% to −0.18%)	−0.21% (−0.26% to −0.16%)
Female	143552718.8 (111560054.71 to 184302641.11)	17129.45 (13404.39 to 21731.64)	62203604.22 (51715024.01 to 71947957.26)	7611.84 (6325.61 to 8809.85)	23768.21 (10672.49 to 45799.2)	2.91 (1.3 to 5.58)	−0.38% (−0.44% to −0.32%)	−0.23% (−0.28% to −0.19%)	−0.22% (−0.27% to −0.17%)
Male	162676250.69 (125429772.5 to 213732608.88)	17818.25 (13926.2 to 22908.35)	69765417.96 (58061172.08 to 81101867.55)	7863.97 (6569.35 to 9100.68)	26641.22 (11993.59 to 51006.55)	3 (1.34 to 5.72)	−0.33% (−0.39% to −0.27%)	−0.22% (−0.28% to −0.17%)	−0.20% (−0.26% to −0.15%)
Southeast Asia									
Both	122523198.04 (90614550.47 to 178330864.89)	20366.78 (15172.99 to 29250.65)	52482821.31 (43544138.38 to 62496935.94)	8898.23 (7377.69 to 10547.34)	20102.07 (8637.67 to 39940.89)	3.41 (1.47 to 6.76)	0.01% (0.00% to 0.03%)	−0.16% (−0.18% to −0.13%)	−0.14% (−0.17% to −0.12%)
Female	59491491.94 (43891887.14 to 86516137.1)	20368.45 (15156.35 to 29229.7)	25481527.47 (21175768.27 to 30270422.51)	8899.14 (7413.65 to 10532.31)	9756.63 (4171.91 to 19475.8)	3.41 (1.46 to 6.79)	0.02% (0.00% to 0.03%)	−0.16% (−0.18% to −0.13%)	−0.14% (−0.17% to −0.12%)
Male	63031706.1 (46663817.74 to 91814727.79)	20365.21 (15189.88 to 29270.43)	27001293.84 (22390633.95 to 32137871.8)	8897.37 (7373.07 to 10530.04)	10345.44 (4465.77 to 20465.09)	3.41 (1.47 to 6.73)	0.01% (0.00% to 0.02%)	−0.16% (−0.19% to −0.13%)	−0.14% (−0.17% to −0.12%)
Australasia									
Both	4204976.97 (2982422.95 to 5271102.07)	20188.03 (14372.51 to 25108.85)	1644455.61 (1382283.77 to 1883117.32)	8145.16 (6853.38 to 9346.44)	629.05 (268.82 to 1261.97)	3.12 (1.33 to 6.27)	0.01% (−0.51% to 0.53%)	0.85% (0.33% to 1.36%)	0.85% (0.33% to 1.36%)
Female	2055374.49 (1422878.37 to 2574303.8)	20316.7 (14215.68 to 25304.76)	786942.18 (653190.65 to 904635.96)	8019.66 (6644.12 to 9218.18)	300.81 (129.17 to 604.77)	3.07 (1.31 to 6.16)	0.04% (−0.49% to 0.57%)	0.86% (0.34% to 1.39%)	0.87% (0.34% to 1.40%)
Male	2149602.48 (1527149.31 to 2707093.74)	20066.18 (14388.56 to 25117.95)	857513.43 (714072.16 to 980269.24)	8263.79 (6861.55 to 9478.77)	328.24 (139.97 to 651.71)	3.16 (1.35 to 6.29)	−0.02% (−0.54% to 0.50%)	0.83% (0.33% to 1.34%)	0.83% (0.32% to 1.33%)
Oceania									
Both	3529853.23 (2584907.12 to 4942758.98)	20143.55 (14576.93 to 28580.36)	1596400.12 (1308960.7 to 1901283.03)	8914.78 (7279.21 to 10654.73)	609.77 (263.87 to 1228.79)	3.41 (1.47 to 6.87)	0.10% (0.07% to 0.14%)	−0.13% (−0.17% to −0.09%)	−0.12% (−0.16% to −0.08%)
Female	1669615.72 (1210372.32 to 2334728.56)	19966.1 (14308.1 to 28332.52)	766817.48 (625604.22 to 912375.15)	8970.32 (7277.6 to 10747.4)	293.11 (127.42 to 597.84)	3.43 (1.49 to 7.01)	0.08% (0.05% to 0.11%)	−0.12% (−0.16% to −0.08%)	−0.11% (−0.15% to −0.08%)
Male	1860237.51 (1361529.05 to 2660807.86)	20304.48 (14717.89 to 29383.01)	829582.64 (671020.63 to 996876.87)	8864.06 (7181 to 10688)	316.66 (134.2 to 626.39)	3.38 (1.43 to 6.69)	0.13% (0.09% to 0.16%)	−0.14% (−0.19% to −0.09%)	−0.13% (−0.18% to −0.08%)
Central Europe									
Both	12389586.77 (8979650.36 to 18026422.74)	20299.17 (14961.56 to 28722.79)	5297643.75 (4368234.51 to 6294003.37)	8918.16 (7373.69 to 10550.01)	2036.37 (869.83 to 3917.35)	3.43 (1.47 to 6.58)	−0.32% (−0.39% to −0.25%)	−0.43% (−0.49% to −0.38%)	−0.42% (−0.48% to −0.36%)
Female	5964265.22 (4373564.75 to 8604281.45)	20152.74 (14992.02 to 28333.91)	2551032.82 (2100023.26 to 3040754.79)	8844.4 (7291.72 to 10472.38)	980.69 (417.43 to 1902.63)	3.4 (1.45 to 6.59)	−0.32% (−0.39% to −0.25%)	−0.45% (−0.51% to −0.39%)	−0.44% (−0.50% to −0.38%)
Male	6425321.56 (4598132.31 to 9422141.28)	20436.96 (14864.99 to 29087.97)	2746610.93 (2268044.92 to 3245867.54)	8987.88 (7451.22 to 10559.42)	1055.68 (450.7 to 2034.41)	3.45 (1.48 to 6.65)	−0.31% (−0.38% to −0.24%)	−0.42% (−0.47% to −0.36%)	−0.40% (−0.45% to −0.35%)
Eastern Europe									
Both	26420072.56 (19463536.58 to 38044622.29)	20737.53 (15,482.44 to 29,309.72)	10,758,608.27 (8,970,359.02 to 12,840,777.36)	8,931.26 (7,458.36 to 10,556.61)	4,133.36 (1,755.68 to 7,897.46)	3.43 (1.47 to 6.55)	−0.01% (−0.02% to 0.01%)	0.04% (0.00% to 0.07%)	0.05% (0.01% to 0.09%)
Female	12,854,157.11 (9,466,605.28 to 18,456,066.12)	20,733.59 (15,546.09 to 29,161.14)	5,219,161.54 (4,347,229.78 to 6,209,934.48)	8,903.93 (7,435.93 to 10,519.66)	2,004.84 (856.02 to 3,855.6)	3.42 (1.48 to 6.56)	−0.01% (−0.02% to −0.01%)	0.03% (−0.01% to 0.06%)	0.04% (0.00% to 0.08%)
Male	13,565,915.45 (9,984,190.17 to 19,593,521.93)	20,741.24 (15,524.45 to 29,393.07)	5,539,446.73 (4,596,388.96 to 6,619,245.56)	8,957.14 (7,442.75 to 10,600.27)	2,128.51 (899.84 to 4,043.09)	3.44 (1.47 to 6.54)	0.00% (−0.02% to 0.02%)	0.05% (0.01% to 0.08%)	0.06% (0.02% to 0.10%)
Western Europe									
Both	33,251,890.96 (24,176,813.95 to 45,855,904.25)	13,957.99 (10,274.55 to 18,969.05)	11,321,247.63 (8,883,702.31 to 14,453,635.76)	4,838.87 (3,811.14 to 6,135.47)	4,341.56 (1,829.32 to 8,786.57)	1.86 (0.78 to 3.76)	−0.30% (−0.41% to −0.19%)	−0.76% (−0.92% to −0.59%)	−0.75% (−0.92% to −0.58%)
Female	15,791,094.43 (11,605,095.52 to 21,921,710.31)	13,605.73 (10,128.31 to 18,622.22)	5,396,179.85 (4,186,382.36 to 6,890,611.48)	4,732.05 (3,705.56 to 5,992.64)	2,067.6 (881.43 to 4,153.93)	1.81 (0.77 to 3.65)	−0.29% (−0.40% to −0.18%)	−0.74% (−0.91% to −0.58%)	−0.74% (−0.91% to −0.57%)
Male	17,460,796.53 (12,525,795.87 to 23,872,715.39)	14,292.66 (10,400.64 to 19,298.49)	5,925,067.78 (4,655,834.72 to 7,555,146.8)	4,940.41 (3,892.91 to 6,257.5)	2,273.96 (947.85 to 4,593.02)	1.9 (0.79 to 3.83)	−0.30% (−0.42% to −0.19%)	−0.76% (−0.93% to −0.60%)	−0.76% (−0.93% to −0.60%)
Andean Latin America									
Both	11,903,981.52 (9,089,490.76 to 15,459,058.21)	18,898.13 (14,474.26 to 24,419.09)	4,929,319.7 (4,095,778.94 to 5,798,201.64)	7,867.51 (6,534.84 to 9,241.53)	1,885.71 (804.96 to 3,670.15)	3.01 (1.29 to 5.85)	0.14% (0.10% to 0.17%)	−0.31% (−0.34% to −0.28%)	−0.30% (−0.34% to −0.27%)
Female	5,588,347.78 (4,293,219.7 to 7,050,684.83)	18,296.03 (14,068.69 to 23,028.07)	2,375,919.94 (1,975,300.27 to 2,793,887.73)	7,810.33 (6,492.94 to 9,186.68)	907.95 (393.23 to 1,749.18)	2.98 (1.29 to 5.75)	0.12% (0.06% to 0.17%)	−0.30% (−0.33% to −0.27%)	−0.30% (−0.33% to −0.26%)
Male	6,315,633.74 (4,603,395.22 to 8,311,305.95)	19,461.78 (14,233.12 to 25,481.59)	2,553,399.76 (2,110,327.33 to 3,021,017.95)	7,921.56 (6,556.59 to 9,356.31)	977.75 (417.2 to 1,937.78)	3.03 (1.3 to 6.01)	0.15% (0.12% to 0.19%)	−0.32% (−0.35% to −0.28%)	−0.31% (−0.34% to −0.27%)
Central Latin America									
Both	38,440,509.78 (29,951,191.05 to 48,192,702.86)	17,455.34 (13,711.31 to 21,568.7)	15,457,774.94 (12,878,285.07 to 18,043,270.58)	7,150.59 (5,944.43 to 8,312.18)	5,937.27 (2,662.92 to 11,277.06)	2.75 (1.23 to 5.2)	−0.24% (−0.32% to −0.17%)	0.09% (0.03% to 0.14%)	0.09% (0.04% to 0.15%)
Female	18,739,531.36 (14,557,234.9 to 23,345,377.72)	17,325.9 (13,559.52 to 21,400.87)	7,591,913.69 (6,321,149.02 to 8,809,711.17)	7,150.53 (5,969.62 to 8,291.98)	2,915.27 (1,303.57 to 5,564.77)	2.75 (1.23 to 5.22)	−0.24% (−0.32% to −0.17%)	0.08% (0.02% to 0.14%)	0.09% (0.03% to 0.15%)
Male	19,700,978.43 (15,385,752.98 to 24,878,713.94)	17,580.41 (13,808.67 to 22,003.01)	7,865,861.25 (6,526,995.33 to 9,301,775.94)	7,150.65 (5,925.09 to 8,425.2)	3,022 (1,338.08 to 5,714.68)	2.75 (1.22 to 5.18)	−0.24% (−0.32% to −0.17%)	0.09% (0.04% to 0.13%)	0.10% (0.05% to 0.15%)
High-income North America									
Both	38,535,114.53 (27,410,640.43 to 49,145,560.96)	17,066.19 (12,243.34 to 21,614.06)	13,548,913.22 (10,672,124.85 to 16,657,394.08)	6,131.77 (4,836.37 to 7,495.01)	5,193.42 (2,152.75 to 10,123.94)	2.35 (0.98 to 4.58)	0.27% (0.14% to 0.41%)	0.21% (−0.16% to 0.58%)	0.20% (−0.16% to 0.57%)
Female	18,693,997.54 (13,282,422.86 to 23,863,080.79)	16,932.96 (12,109.06 to 21,501.03)	6,510,680.28 (5,114,767.62 to 8,019,184.07)	6,022.93 (4,765.24 to 7,378.87)	2,495.78 (1,041.4 to 4,829.96)	2.31 (0.97 to 4.47)	0.29% (0.15% to 0.43%)	0.25% (−0.11% to 0.62%)	0.25% (−0.11% to 0.61%)
Male	19,841,116.99 (14,192,927.32 to 25,458,855.85)	17,193.55 (12,406.91 to 21,839.56)	7,038,232.94 (5,557,428.02 to 8,603,688.15)	6,235.81 (4,919.65 to 7,587.98)	2,697.65 (1,117.4 to 5,293.98)	2.39 (0.99 to 4.69)	0.26% (0.12% to 0.39%)	0.16% (−0.21% to 0.54%)	0.16% (−0.21% to 0.53%)
Southern Latin America									
Both	9,840,834.32 (6,991,458.46 to 13,655,068.08)	18,955.35 (13,692.37 to 25,968.34)	3,723,136.03 (2,929,915.61 to 4,674,994.91)	7,412.4 (5,884.43 to 9,239.84)	1,429.34 (583.93 to 2,864.38)	2.85 (1.18 to 5.71)	−0.02% (−0.07% to 0.04%)	−0.05% (−0.15% to 0.05%)	−0.05% (−0.14% to 0.05%)
Female	4,782,570.99 (3,335,208.29 to 6,538,634.3)	18,804.86 (13,335.22 to 25,406.67)	1,788,759.49 (1,393,920.15 to 2,247,935.7)	7,264.37 (5,725.66 to 9,046.48)	686.38 (285.92 to 1,356.2)	2.79 (1.17 to 5.53)	−0.06% (−0.10% to −0.01%)	−0.07% (−0.17% to 0.03%)	−0.07% (−0.17% to 0.03%)
Male	5,058,263.33 (3,578,361.19 to 7,200,053.44)	19,099.99 (13,710.01 to 26,629.2)	1,934,376.54 (1,542,035.29 to 2,435,924.73)	7,554.34 (6,018.62 to 9,424.26)	742.96 (314.2 to 1,466.3)	2.9 (1.23 to 5.7)	0.02% (−0.03% to 0.08%)	−0.03% (−0.13% to 0.06%)	−0.03% (−0.12% to 0.06%)
Tropical Latin America									
Both	35,472,271.88 (25,338,048.02 to 51,290,262.16)	20,346.12 (14,535.28 to 29,159.69)	13,360,204.43 (10,524,334.69 to 16,327,212.94)	7,705.41 (6,076.1 to 9,396)	5,100.87 (2,127.35 to 10,357.34)	2.94 (1.23 to 5.97)	0.15% (0.03% to 0.27%)	0.03% (−0.09% to 0.14%)	0.04% (−0.08% to 0.15%)
Female	17,380,123.16 (12,369,037.67 to 25,295,327.19)	20,380.89 (14,571.32 to 29,333.15)	6,513,273.02 (5,140,386.68 to 7,937,000.23)	7,684.21 (6,068.59 to 9,342.41)	2,484.08 (1,025.31 to 5,049.01)	2.93 (1.21 to 5.95)	0.14% (0.01% to 0.26%)	0.01% (−0.10% to 0.13%)	0.02% (−0.09% to 0.14%)
Male	18,092,148.72 (12,861,526.55 to 26,146,825.07)	20,312.72 (14,522.25 to 29,109.46)	6,846,931.41 (5,381,232.78 to 8,352,752.74)	7,725.66 (6,075.26 to 9,414.17)	2,616.79 (1,088.95 to 5,256.5)	2.95 (1.23 to 5.93)	0.16% (0.05% to 0.27%)	0.04% (−0.08% to 0.15%)	0.05% (−0.07% to 0.16%)
Caribbean									
Both	6,900,574.05 (5,333,356.94 to 8,543,403.21)	17,357.91 (13,436.75 to 21,465.21)	3,061,941.47 (2,579,575.9 to 3,549,705.13)	7,760.8 (6,539.68 to 8,983.62)	1,172.18 (530.34 to 2,312.3)	2.97 (1.35 to 5.87)	−0.12% (−0.21% to −0.03%)	−0.10% (−0.14% to −0.06%)	−0.09% (−0.13% to −0.05%)
Female	3,376,209.08 (2,601,689.99 to 4,197,217.1)	17,299.81 (13,371.8 to 21,420.72)	1,502,921.96 (1,269,288.8 to 1,741,231.98)	7,762.29 (6,554.75 to 8,981.11)	574.31 (259.54 to 1,133.48)	2.97 (1.34 to 5.86)	−0.15% (−0.26% to −0.04%)	−0.11% (−0.15% to −0.06%)	−0.10% (−0.14% to −0.05%)
Male	3,524,364.98 (2,719,435.88 to 4,366,221.73)	17,414.07 (13,489.34 to 21,518.84)	1,559,019.51 (1,319,359.47 to 1,797,583.79)	7,759.36 (6,560.88 to 8,941)	597.87 (271.03 to 1,183.76)	2.98 (1.35 to 5.89)	−0.09% (−0.18% to 0.00%)	−0.10% (−0.14% to −0.06%)	−0.08% (−0.12% to −0.04%)
North Africa and Middle East									
Both	104,324,034.12 (80,198,383.83 to 129,932,689.1)	16,170.92 (12,510.74 to 20,082.24)	47,656,406.08 (39,924,948.59 to 55,589,059.04)	7,446.43 (6,242.55 to 8,668.07)	18,258.4 (8,393.62 to 35,367)	2.85 (1.31 to 5.52)	−0.78% (−0.89% to −0.68%)	−0.25% (−0.28% to −0.22%)	−0.24% (−0.28% to −0.21%)
Female	51,040,341.78 (39,027,772.41 to 63,471,537.64)	16,313.87 (12,547.6 to 20,230.55)	23,373,384.98 (19,626,502.12 to 27,471,455.77)	7,527.96 (6,330.93 to 8,854.44)	8,953.87 (4,078.01 to 17,413.52)	2.88 (1.31 to 5.6)	−0.76% (−0.88% to −0.64%)	−0.20% (−0.22% to −0.18%)	−0.20% (−0.22% to −0.18%)
Male	53,283,692.34 (41,265,628.65 to 66,402,965.06)	16,036.39 (12,452.36 to 19,915.97)	24,283,021.1 (20,324,945.18 to 28,094,735.73)	7,369.71 (6,163.86 to 8,503.72)	9,304.52 (4,208.36 to 17,944.47)	2.82 (1.27 to 5.44)	−0.81% (−0.93% to −0.69%)	−0.29% (−0.34% to −0.25%)	−0.29% (−0.33% to −0.24%)
Central Sub-Saharan Africa									
Both	28,086,483.83 (18,578,865.77 to 37,440,315.91)	13,581.43 (8,982.29 to 18,153.81)	12,096,947.77 (9,024,829.07 to 15,066,625.78)	5,836.05 (4,359.13 to 7,266.19)	4,622.45 (2,037.25 to 9,551.27)	2.23 (0.98 to 4.61)	−1.23% (−1.39% to −1.08%)	−0.92% (−1.04% to −0.80%)	−0.91% (−1.03% to −0.79%)
Female	13,793,148.6 (9,085,477.46 to 18,416,739.77)	13,493.05 (8,875.53 to 18,026.7)	5,968,736.05 (4,445,303.39 to 7,537,208.2)	5,828.53 (4,345.19 to 7,360.73)	2,282.29 (969.91 to 4,644.87)	2.23 (0.95 to 4.54)	−1.21% (−1.42% to −0.99%)	−0.93% (−1.09% to −0.77%)	−0.92% (−1.08% to −0.76%)
Male	14,293,335.23 (9,545,247.07 to 19,086,257.93)	13,668.02 (9,138.44 to 18,251.45)	6,128,211.72 (4,536,434.2 to 7,566,354.11)	5,843.35 (4,334.23 to 7,219.71)	2,340.17 (1,037.7 to 4,932.74)	2.23 (0.99 to 4.7)	−1.25% (−1.47% to −1.04%)	−0.91% (−1.09% to −0.74%)	−0.90% (−1.07% to −0.72%)
Eastern Sub-Saharan Africa									
Both	104,116,834.98 (81,665,739.22 to 131,785,477.05)	16,818.21 (13,182.33 to 21,313.33)	43,782,769.2 (36,046,664.25 to 50,567,850.33)	7,045.12 (5,804.6 to 8,137.05)	16,708.92 (7,402.48 to 32,571.45)	2.69 (1.19 to 5.24)	−0.48% (−0.59% to −0.37%)	−0.48% (−0.57% to −0.39%)	−0.46% (−0.55% to −0.37%)
Female	50,863,860.73 (39,762,427.74 to 64,779,667.49)	16,680.53 (13,035.54 to 21,263.49)	21,259,173.77 (17,607,378.82 to 24,631,866.71)	6,949.24 (5,755.91 to 8,054.1)	8,115.12 (3,606.68 to 15,812.27)	2.65 (1.18 to 5.17)	−0.46% (−0.58% to −0.34%)	−0.45% (−0.55% to −0.35%)	−0.43% (−0.53% to −0.33%)
Male	53,252,974.25 (41,860,275.46 to 67,220,733.76)	16,951.73 (13,307.33 to 21,421.43)	22,523,595.43 (18,544,085.99 to 25,982,680.33)	7,137.93 (5,878.47 to 8,227.02)	8,593.8 (3,783.25 to 16,724.62)	2.72 (1.2 to 5.3)	−0.50% (−0.62% to −0.38%)	−0.51% (−0.60% to −0.42%)	−0.49% (−0.58% to −0.40%)
Southern Sub-Saharan Africa									
Both	15,319,127.79 (11,308,422.05 to 19,692,964.04)	18,238.4 (13,484.31 to 23,381.99)	5,783,521.12 (4,539,832.65 to 7,229,862.75)	6,959.22 (5,477.83 to 8,688.31)	2,212.56 (928.19 to 4,402)	2.66 (1.12 to 5.3)	0.19% (0.14% to 0.24%)	0.39% (0.26% to 0.52%)	0.40% (0.26% to 0.53%)
Female	7,484,323.18 (5,665,394.14 to 9,407,462)	17,976.83 (13,641.19 to 22,535.98)	2,862,755.17 (2,249,681.84 to 3,565,883.01)	6,951.11 (5,472.5 to 8,648.31)	1,095.6 (460.26 to 2,178.87)	2.66 (1.12 to 5.3)	0.19% (0.13% to 0.24%)	0.38% (0.24% to 0.52%)	0.39% (0.25% to 0.53%)
Male	7,834,804.61 (5,668,600.55 to 10,361,486.11)	18,495.6 (13,397.99 to 24,381.4)	2,920,765.95 (2,288,141.34 to 3,666,685.99)	6,967.22 (5,464.84 to 8,728.87)	1,116.96 (465.3 to 2,218.55)	2.66 (1.11 to 5.3)	0.20% (0.15% to 0.25%)	0.40% (0.27% to 0.53%)	0.40% (0.28% to 0.53%)
Western Sub-Saharan Africa									
Both	126,472,257.62 (96,989,928.73 to 160,153,636.42)	16,945.19 (12,928.71 to 21,594.67)	52,295,133.43 (41,821,168.95 to 62,995,112.61)	6,924.38 (5,539.39 to 8,340.24)	19,940.07 (8,483.52 to 38,806.63)	2.64 (1.12 to 5.13)	−0.37% (−0.44% to −0.31%)	0.05% (−0.03% to 0.13%)	0.06% (−0.02% to 0.14%)
Female	62,264,799.04 (47,852,531.21 to 78,807,212.27)	16,811.85 (12,883.01 to 21,357.34)	25,939,521.12 (20,827,411.62 to 31,209,652)	6,934.17 (5,570.28 to 8,349.42)	9,889.68 (4,226.84 to 19,187.43)	2.64 (1.13 to 5.13)	−0.34% (−0.41% to −0.27%)	0.02% (−0.06% to 0.11%)	0.04% (−0.05% to 0.12%)
Male	64,207,458.58 (48,883,566.25 to 81,655,542.28)	17,078.61 (12,927.55 to 21,892.34)	26,355,612.31 (21,031,016.74 to 31,759,092.22)	6,914.97 (5,512.85 to 8,343.31)	10,050.39 (4,256.68 to 19,626.53)	2.64 (1.12 to 5.14)	−0.41% (−0.47% to −0.34%)	0.07% (−0.01% to 0.15%)	0.09% (0.01% to 0.17%)

Values in parentheses represent 95% uncertainty intervals.

ASIR, age-standardized incidence rate; ASPR, age-standardized prevalence rate; ASYR, age-standardized YLD rate; ASR, age-standardized rate (per 100,000 population).

#### High-income Asia-Pacific and Western Europe

These regions showed the lowest burden of dental caries, with incidence and prevalence rates significantly lower than in other regions. The declining EAPC for these regions indicates effective and sustained public health measures.

#### South Asia and sub-Saharan Africa

South Asia and Sub-Saharan Africa had high incidence and ASYR, reflecting deficiencies in oral health services and weak public health infrastructure that hinder effective caries prevention. For example, East Sub-Saharan Africa had a prevalence ASR of 7,045.12 per 100,000, with an ASYR of 2.69.

### Sex differences

Across most regions, the incidence and prevalence rates of dental caries in males were consistently slightly higher than those in females. For example, in East Asia, the ASR for males was 19,524.28 per 100,000, compared to 19,548.95 per 100,000 for females, indicating a slight sex disparity. Some regions, such as South Asia and Western Europe, showed a more pronounced decline in EAPC for females, suggesting these areas may focus more on oral health education for female children.

### Trends in dental caries in primary teeth from 1990 to 2040

#### Incidence trend

**Figure 3:**
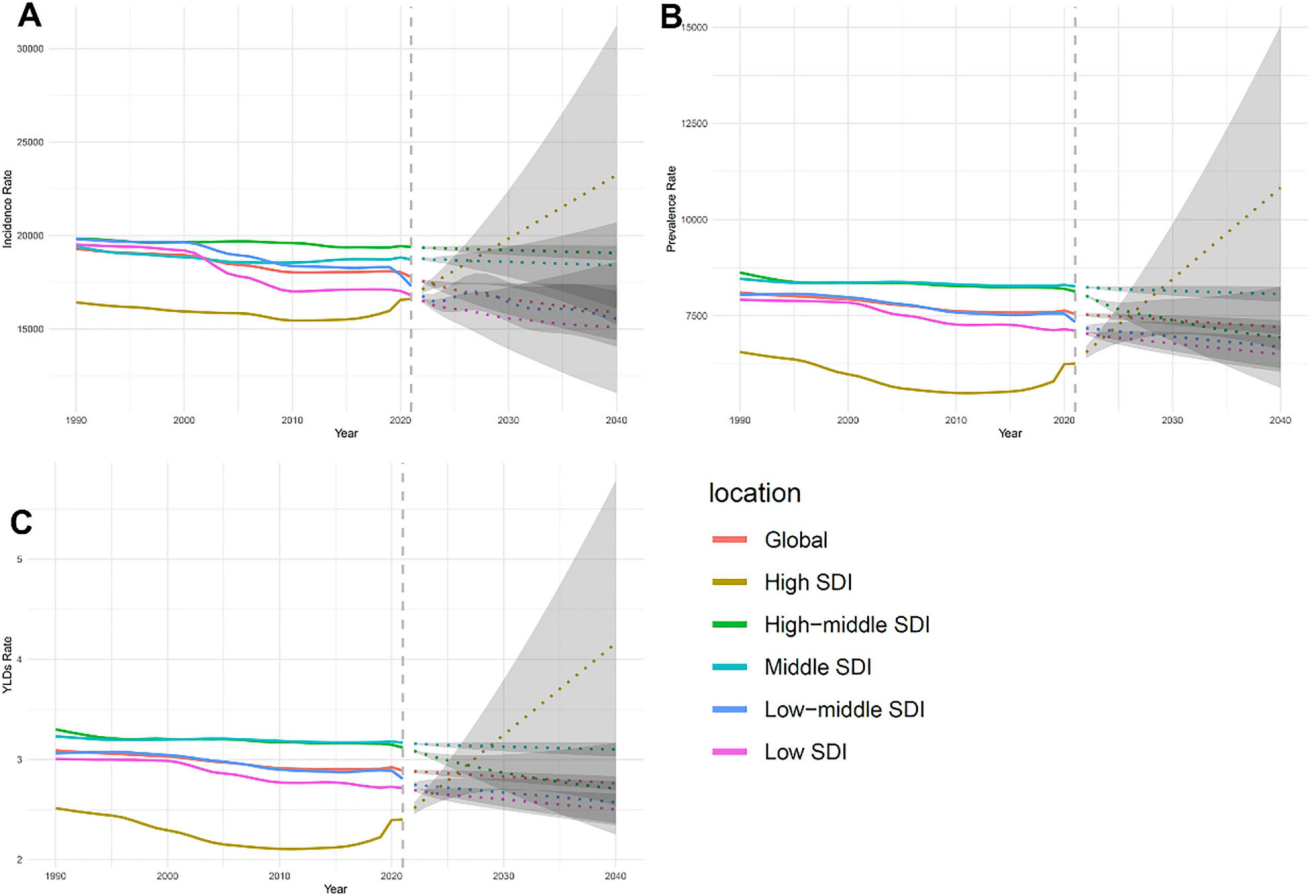
Trends in incidence **(A)**, prevalence **(B)**, and age-standardized years lived with disability (YLD) rate (ASYR) **(C)** of dental caries in primary teeth from 1990 to 2040, highlighting variations across different socio-demographic index (SDI) regions.

The global incidence of dental caries in primary teeth decreased from 1990 to 2020. However, predictions beyond 2020 indicate that incidence may rebound, especially in low, medium, and low-middle SDI regions. These regions had large uncertainty intervals, indicating unpredictability in future trends (Figure 3A).

#### ASYR trend

The ASYR showed a decreasing trend globally, suggesting improvements in treatment and management over the past three decades. However, differences between SDI regions remained, with high SDI regions showing significantly lower ASYR compared to low SDI regions (Figure 3B).

#### Prevalence trend

The prevalence rate showed a similar decline, especially in high SDI regions. However, the prevalence rate in low, medium, and low-middle SDI regions showed considerable potential fluctuations, possibly related to limited healthcare access and poor oral health infrastructure (Figure 3C).

### Overall trends and regional disparities

The results from Figure 3 indicate significant regional disparities in the burden of dental caries in primary teeth. High SDI regions showed significant reductions and lower predicted variations in incidence, prevalence, and ASYR, suggesting that interventions have been effective. In contrast, low and medium SDI regions are predicted to experience more variability and challenges in controlling caries rates.

## Discussion

The findings of this study provide important insights into the global burden of dental caries in primary teeth, highlighting significant variations in incidence, prevalence, and ASYR across different SDI regions, age groups, and sexes. These disparities underscore the need for targeted public health strategies to address dental caries in specific populations.

The declining trend in the incidence, prevalence, and ASYR of dental caries in primary teeth from 1990 to 2021, as indicated by the EAPC, suggests a modest improvement in the global burden of this condition. However, the limited reduction highlights the ongoing challenges in preventing and managing dental caries worldwide. The reduction was particularly modest in low and medium SDI regions, where socio-economic factors, inadequate healthcare infrastructure, and limited access to oral health services may hinder effective prevention and treatment (19). These findings suggest that despite global efforts to improve oral health, the existing interventions are insufficient to address the needs of under-resourced populations (20).

Differences across SDI regions were prominent, with high SDI regions exhibiting the lowest burden of dental caries, both in terms of incidence and prevalence. This can likely be attributed to better access to healthcare, improved oral hygiene practices, and greater awareness of preventive measures in these regions (21). In contrast, medium and low SDI regions showed higher incidence and prevalence rates, with medium SDI regions experiencing a peak in both metrics (19). The higher burden in these regions may be related to the transitional socio-economic conditions that increase susceptibility to dental caries, such as changing diets high in sugar and inadequate public health measures to counteract these risks. The findings also suggest that high SDI regions have benefited from sustained public health interventions, while medium and low SDI regions require more focused efforts to bridge the gap in oral health outcomes (22).

Sex differences in dental caries were also evident, with males consistently showing slightly higher incidence and prevalence rates compared to females, although ASYR showed no significant sex difference. This disparity may be linked to differences in dietary habits, oral hygiene practices, and health-seeking behaviors between males and females (23). For example, males may be less likely to engage in preventive oral health behaviors, such as regular brushing and dental visits, which could contribute to the observed higher rates of dental caries (24). These findings indicate a need for sex-specific health education and interventions that address the unique behaviors and risks associated with dental caries in males.

Age-specific analysis revealed that children aged 2–9 years were the most burdened by dental caries, particularly those aged 5–9 years. The high incidence and prevalence in this age group are likely due to the complete eruption of primary teeth and the challenges associated with maintaining proper oral hygiene during early childhood. The findings emphasize the importance of focusing public health interventions on younger children to reduce the burden of dental caries. Effective strategies could include early education for parents and caregivers on the importance of oral hygiene, reducing sugar intake, and increasing access to preventive dental care (25). Moreover, public health policies should prioritize integrating oral health into broader child health programs to ensure that preventive measures are accessible to all children, particularly in vulnerable communities (26).

The trends observed from 1990 to 2040 indicate that, while progress has been made in reducing the burden of dental caries in high SDI regions, challenges remain in low and medium SDI regions. The predicted rebound in incidence rates in these regions after 2020 underscores the importance of continuous and adaptable public health efforts. Factors such as limited healthcare access, socio-economic instability, and lack of awareness about oral health are likely contributing to the uncertainty and potential increase in caries incidence. These findings suggest that sustained investment in healthcare infrastructure, community-based oral health initiatives, and school-based preventive programs are crucial for mitigating the burden of dental caries in these populations (21).

The results of this study illustrate the importance of focusing public health interventions on younger children to reduce the burden of dental caries. Preventive oral health programmes in schools have been shown to be very effective in instilling proper oral hygiene practices and dietary awareness at an early age (8, 10, 11). Collaboration between schools, dental hospitals and public health organisations can enhance regular dental check-ups, supervised brushing and interactive health education to reduce the risk of dental caries. In addition, educating parents and caregivers, especially in communities with limited awareness of caries risk, about reducing children's sugar intake, balanced nutrition and proper oral hygiene practices can significantly enhance the effectiveness of prevention programmes (10, 11, 15). These approaches not only address immediate risk factors, but also help to develop long-term healthy behaviours in children.

Overall, this study highlights the complex interplay between socio-economic factors, healthcare access, sex, and age in influencing the burden of dental caries in primary teeth. To achieve significant reductions in dental caries globally, particularly in under-resourced regions, public health strategies must be multifaceted and tailored to address the specific needs of different populations. Key recommendations include improving access to dental care, enhancing community-level oral health education, and implementing targeted interventions for high-risk groups such as young children and males. By focusing on these areas, it is possible to make meaningful progress in reducing the global burden of dental caries in primary teeth and improving children's oral health outcomes overall.

## Data Availability

The raw data supporting the conclusions of this article will be made available by the authors, without undue reservation.
